# Association between forearm cortical bone properties and handgrip strength in women with distal radius fractures: A cross-sectional study

**DOI:** 10.1371/journal.pone.0243294

**Published:** 2020-12-03

**Authors:** Seok Woo Hong, Jeong-Hyun Kang, Jong Seop Kim, Hyun Sik Gong

**Affiliations:** 1 Department of Orthopedic Surgery, Kangbuk Samsung Hospital, Sungkyunkwan University School of Medicine, Seoul, Korea (ROK); 2 Clinic of Oral Medicine and Orofacial Pain, Institute of Oral Health Science, Ajou University School of Medicine, Suwon, Gyeonggi-do, Korea (ROK); 3 Department of Orthopedic Surgery, Seoul National University Bundang Hospital, Seoul National University College of Medicine, Seongnam, Gyeonggi-do, Korea (ROK); 4 Department of Orthopedic Surgery, Seoul National University Bundang Hospital, Seoul National University College of Medicine, Seongnam, Gyeonggi-do, Korea (ROK); China University of Mining and Technology, CHINA

## Abstract

**Objectives:**

Mechanical and biochemical bone properties are influenced by muscles. However, the muscle-bone interaction has not been fully elucidated regarding the upper extremities. The objective of the present study was to evaluate the mechanical muscle-bone interaction at the forearm by evaluating the relationship between the properties of three-dimensional (3D) forearm cortical bone models derived from conventional computed tomography (CT) images and handgrip strength (HGS).

**Methods:**

A total of 108 women (mean age, 75.2 ± 9.4 years; range, 62–101 years) with a distal radius fracture who took conventional CT scans for the assessment of the fracture were included in this study. Distal radius 3D models were reconstructed and the average cortical bone density (Cd) and thickness (Ct) of the region of interest (ROI), which might be affected by the forearm flexor muscles, were calculated using a 3D modeling software. Clinical parameters including HGS, lumbar and hip bone mineral densities (BMDs), and other demographic factors were also obtained. A multivariate linear regression analysis was performed to identify relevant factors associated with HGS.

**Results:**

HGS was found to be independently associated with height and Cd, but no significant difference was found between HGS and Ct, age, weight, as well as lumber and hip BMDs.

**Conclusions:**

Cortical bone density might be associated with HGS, which is generated by the forearm flexor muscles. Hence, the mechanical muscle-bone interaction in the upper extremities could be supported by the present study.

## Introduction

Osteoporosis is one of the most important metabolic diseases characterized by decreased bone mass, damaged bone microstructure, and weakened bone strength [1]. Osteoporotic fractures may emerge due to weakened bone strength; therefore many studies have been conducted to figure out an effective method for bone strength reinforcement [2]. Several reports have been revealed regarding the associations between bone strength and cortical bone properties, including cortical bone thickness (Ct) and cortical density (Cd) [3, 4]. Traditionally, Ct and Cd are evaluated using two-dimensional (2D) plain radiographs [5, 6] and/or peripheral quantitative computed tomography (pQCT) [7]. However, 2D plain radiographs cannot provide enough information in association with Cd and have limitations in Ct measurement, including image distortion and the superimposition of skeletal structures. Besides, pQCT is not easily accessible in routine clinical circumstances due to the requirement of special equipment, including a diverse range of monitoring devices, and trained personnel for the interpretation of the pQCT data [8].

In contrast, conventional CT scans are not only able to provide information in association with fracture patterns [9] but also provide additional information on bone properties, which are therefore called “opportunistic osteoporosis CT scans” [10]. Owing to the advances in 3D graphic processing technologies, the measurement of the thickness and density of the cortical bone in the target region of interests (ROI) using CT data has become possible [11, 12]. Specifically, the average Hounsfield Unit (HU) and cortical bone thickness can be automatically calculated using a 3D graphic processing software. The correlations between the areal bone mineral density (aBMD) of the hip and the lumbar and the average HU of the distal ulna were demonstrated by a previous study [13] suggesting the use of HU as a parameter for the evaluation of local BMDs in ROI.

“Muscle-bone interactions” indicate that the two organs interact with each other for function and homeostasis regulation [14]. The muscle-bone interaction is not only able to imply the anatomical relationships but functional connections as well [15]. These interactions could occur locally or distantly via various mechanical stimulations or several biomechanical signals [14, 15]. The load transmission to the bone by the muscles is carried out at their attachment sites [15]. The remodeling capacities of the bone can be influenced by the amount of transmitted load from the contraction of the muscle [14–16].

Handgrip strength (HGS) is one of the most widely used parameters, suggesting systemic overall muscle condition and fragility [17, 18] as well as physical ability and function [19]. HGS is known to be an indicator of the degree of bone metabolism and the occurrence of fractures [20]. HGS is generated by forearm flexor muscles, which are mostly originated from the forearm cortical bones [21]. To the best of our knowledge, sparse studies have been conducted to investigate the focal interactions between the forearm flexor muscle activities and the cortical bone qualities of the radial forearm.

The advancement in 3D graphic processing technology has made the evaluation of the cortical bone quality possible based on conventional CT images, especially in the craniofacial field [11, 22]. The validity and reliability of such an assessment method in association with the measurement of the cortical bone density in the craniofacial field have been shown [11]. However, few studies have investigated the qualities of extremity skeletal cortical bone with the application of this technology [12].

Therefore, the purposes of this study were to evaluate the mechanical muscle-bone interaction at the forearm through the assessment of the relationship between the properties of 3D forearm cortical bone models derived from conventional CT images and HGS in patients with distal radius fracture (DRF) as well as to analyze the properties of the radial forearm cortical bone using a 3D graphic processing software.

## Materials and methods

### Participants

**Fig 1:**
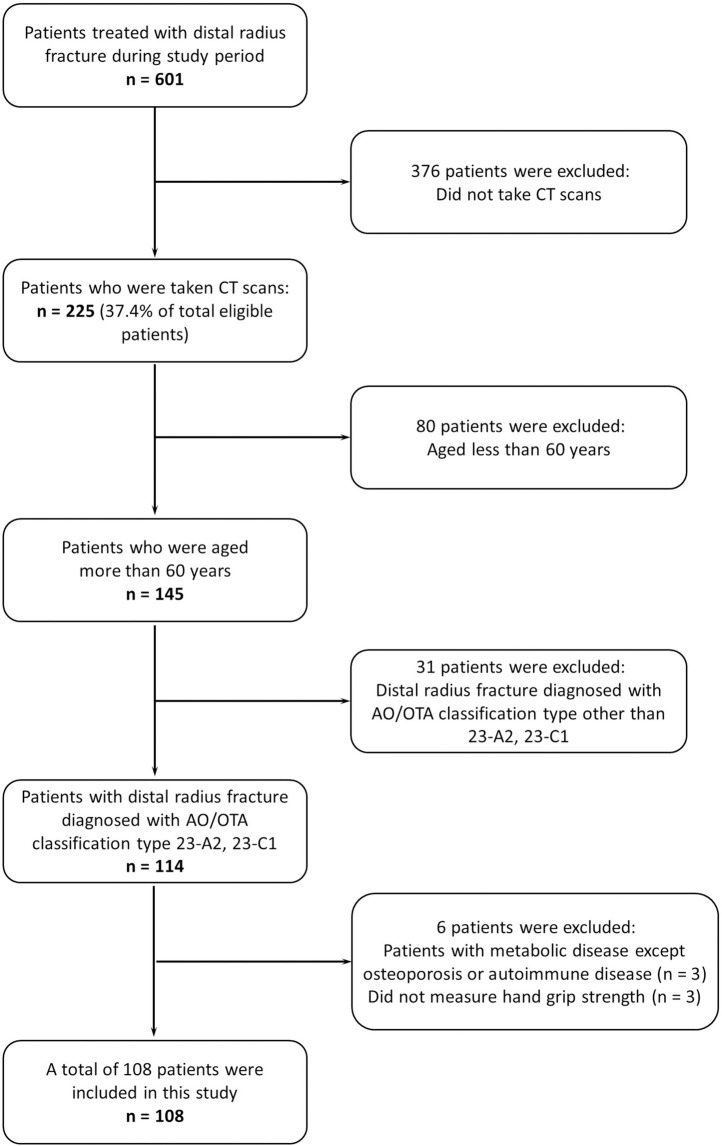
A flow chart of patients included in this study.

This was a single-center retrospective study of 108 female patients (mean age, 75.2 ± 9.4 years; range, 62–101 years) with DRF from a tertiary care hospital using conventional CT, DXA, and clinical records. The inclusion criteria were patients with (1) DRF diagnosed with AO/OTA classification type 23-A2 (Extra-articular, simple or impacted type) and 23-C1 (Complete-articular, simple joint and simple metaphysis type), (2) a conventional wrist CT scan performed immediately after the manual reduction of the fracture, (3) available aBMD data measured within 3 months before or after the injury, (4) HGS measured at the contralateral non-injured side, and (5) being a post-menopausal female over 60 years. Patients with metabolic diseases except for osteoporosis or autoimmune diseases that might affect bony metabolism were excluded from this study (**Fig 1**). All patients included in the present study had unilateral DRF from January 2016 to December 2017. The following demographic and clinical data were compiled through an electronic medical record system: age, affected side and hand dominance, height, and body weight. A pre-examination questionnaire was used for conducting interviews about basic information, including hand dominance and underlying disease. All data investigated in this study were first fully anonymized and subsequently used for analysis. This study was undertaken by following the research protocol approved by the Institutional Review Board of the University Hospital (B-1808/489-107) and the requirement was waived to obtain informed consent.

### Measurements of handgrip strength at the injured side

HGS was measured by a hand dynamometer (Jamar^®^ 5030J1 hydraulic hand dynamometer, Sammons Preston Rolyan, Bolingbrook, IL, USA) at the non-injured contralateral hand at the initial visit to the clinic. The measurement was conducted by a trained clinical research nurse and taken in a sitting position with a 90° of elbow flexion and neutral forearm position [23]. All participants were instructed to perform a test with their maximal grip strength. Each participant was measured three times at intervals of five minutes and the average HGS value was subsequently calculated. The 10% rule was applied for the estimation of the HGS when the dominant hand was identified to be the injured side [24, 25].

### 3D reconstruction of radial forearm cortical bone

Wrist CT images were taken immediately after the closed reduction of the fracture in the emergency department using a 256-slice multi-detector CT scanner (Brilliance iCT 256, Philips Medical Systems, Amsterdam, the Netherlands). The following scanning protocol was used: 120 kVp tube potential; 149 mAs tube current-time product; 128 mm × 0.625 mm section collimation; 0.5 ms rotation time; 0.4 pitch; 180 mm display field of view; pixel size 0.3 mm × 0.3 mm; and 1 mm section thickness. Corrected coronal, sagittal, and axial images of the wrist were saved as Digital Imaging and Communications in Medicine (DICOM) files.

**Fig 2:**
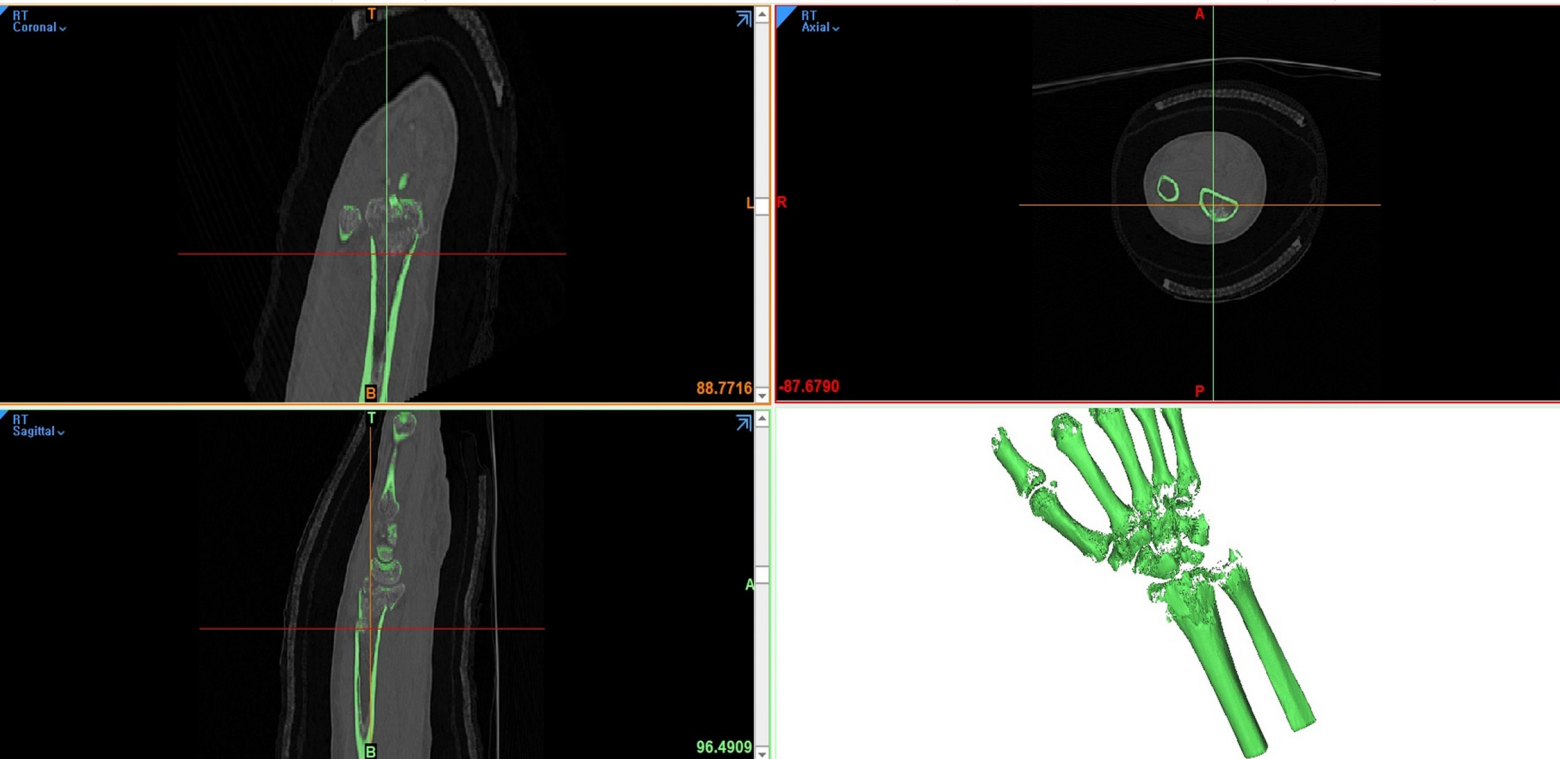
A screenshot of the 3D reconstruction modeling software. Digitalized CT data in DICOM format were imported and the coronal, sagittal, and axial views of the CT data were obtained in Mimics^®^ (Mimics^®^ 22.0, Materialise, Antwerp, Belgium). The green–colored reconstruction image in the right bottom shows a density mask. Voxels with attenuation above 850 HU in CT images converted to density masks in the Mimics^®^ software.

Digitalized CT data in the DICOM files were imported into a 3D reconstruction modeling software (Mimics^®^ 22.0, Materialise, Antwerp, Belgium) (**Fig 2**). HU thresholding technique was used to reconstruct the dense cortical bone of the target site and the attenuation threshold of the dense cortical bone was set to 850 HU [26, 27]. Voxels with attenuation above 850 HU were converted to density masks in the Mimics^®^ software.

### Evaluation of the cortical bone properties (Ct and Cd)

**Fig 3:**
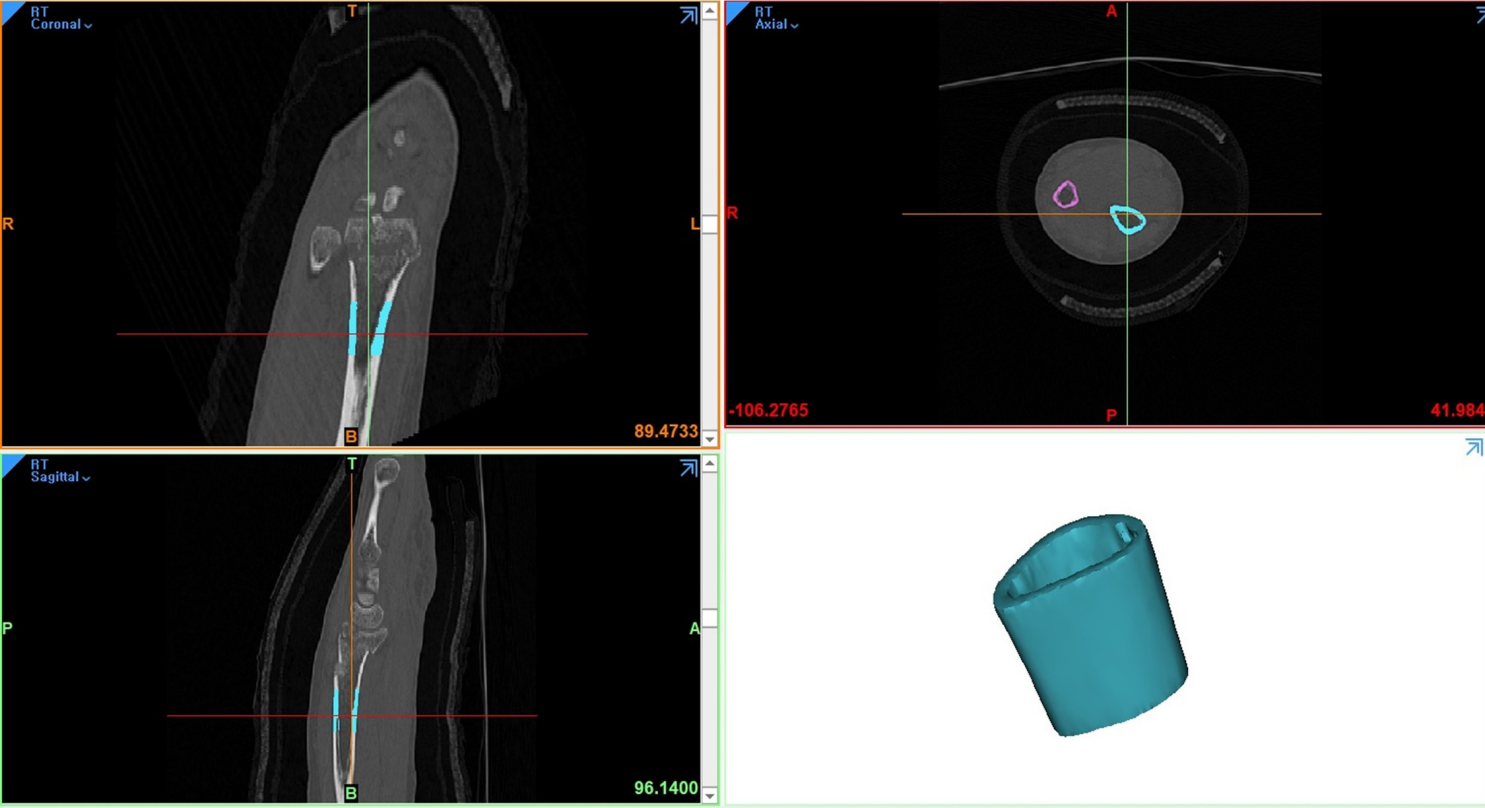
Region of Interests (ROI) of the radial forearm cortical bone. A 2 cm long cylindrical mask was created 3 cm proximal to the lunate fossa of the radius (A blue cylinder). The ROI of the radial forearm cortical bone mask defined.

**Fig 4:**
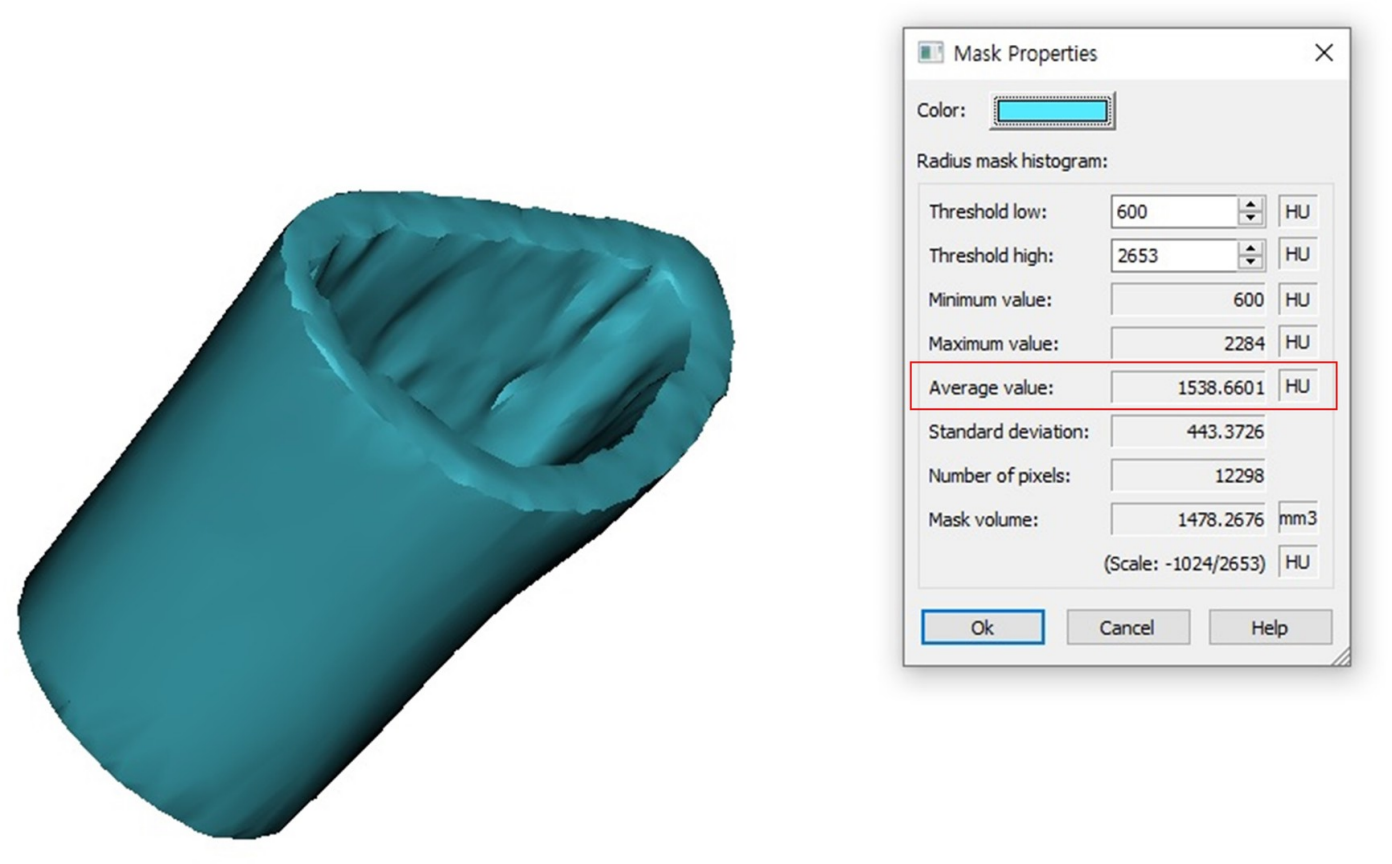
Average Hounsfield Unit (HU) calculation. Automatic calculation of the average HU (Cd of ROI) in the Mimics^®^ software.

**Fig 5:**
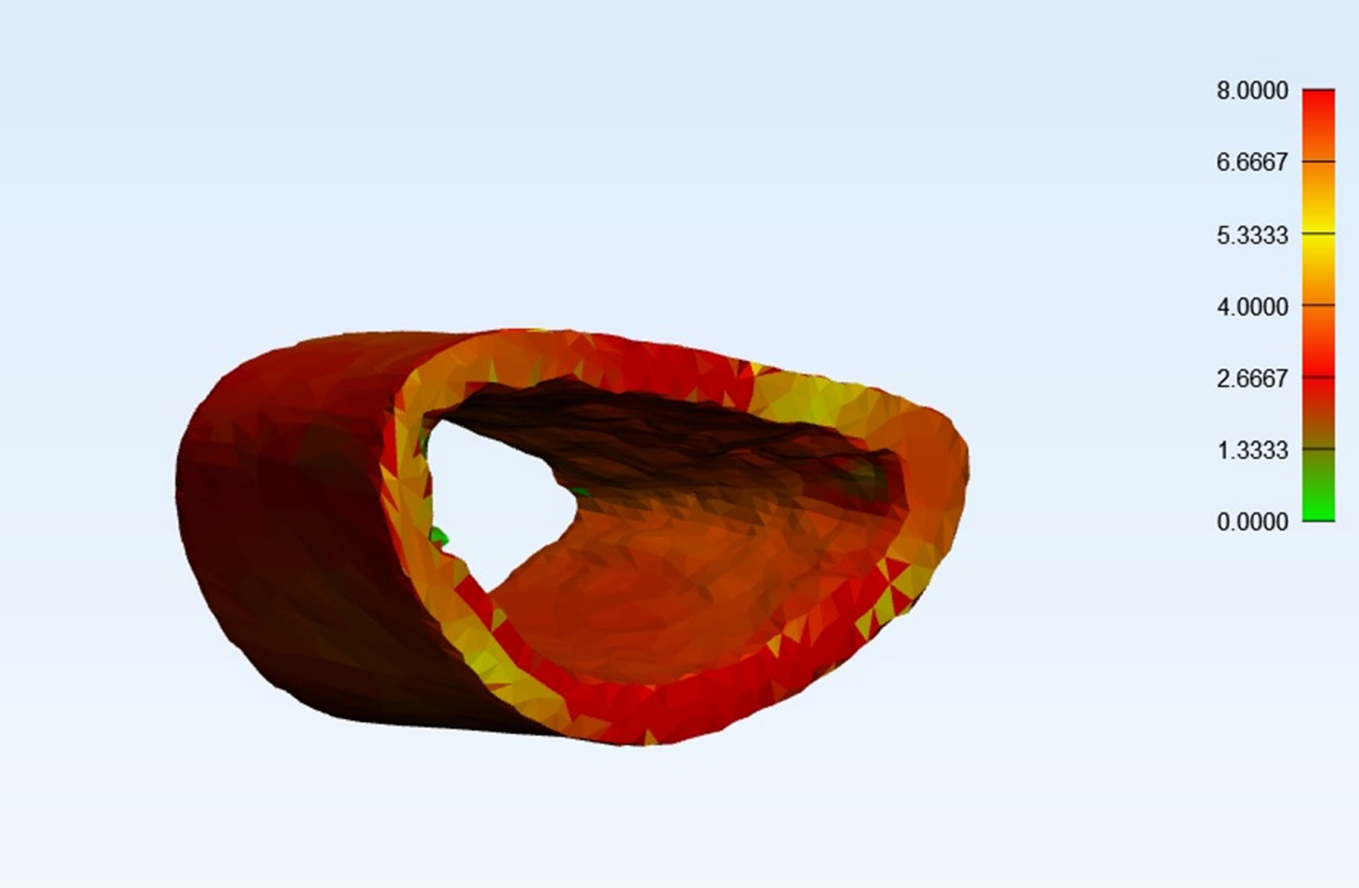
Average cortical thickness calculation. Automatic calculation of the average cortical thickness (Ct of ROI) in the Mimics^®^ software.

Because fractures often affect cortical bone loss, which might lead to low HU measurements [28], the cortical bone properties were determined on the part of the radius that was not affected by the fractures. A 2cm long cylindrical mask was created 3cm proximal to the lunate fossa of the radius. The mask defined the ROI of the radial forearm cortical bone (**Fig 3**). The average HU (Cd) and thickness (Ct) of the ROI was automatically calculated using the Mimics^®^ software (**Figs 4 and 5**).

### Measurements of systemic bone mineral density

Systemic areal bone mineral densities (aBMDs) were evaluated by DXA (Horizon-W; Hologic Inc., Bedford, MA, USA) in the lumbar and the femur areas. The least significant change (g/cm^2^) of aBMDs with a 95% confidence level was 0.015 for the femur neck, 0.006 for the total femur, and 0.009 for the total lumbar spine. All DXA data were obtained within 3 months before or after the injury. The BMD of the femur was measured from the femur neck and the total femur, and the BMD of the spine was measured from the total lumbar spine (L_1_ through L_4_). The results were expressed as the absolute value of aBMD (g/cm^2^).

### Statistical analysis

A power analysis indicated that a sample of 108 participants for a multiple linear regression with 6 main predictors would provide 85% statistical power at a 0.05 significance level with a medium effect size (f^2^ = 0.15). A Shapiro–Wilk normality test was applied and that the data from the present study were normally distributed. Therefore, parametric tests were used. Asian working group for sarcopenia proposed that females with HGS lower than 18 kg have a high risk for sarcopenia [29]. Therefore, the difference of body mass index [BMI, weight (kg) / height^2^ (m^2^)], aBMDs, as well as the Ct and the Cd of the radial forearm bones in patients with HGS lower than 18 kg and those with HGS equal to or higher than 18 kg were determined by independent t-test. The relationship between the HGS and each independent variable (demographic factors, aBMDs, Ct, Cd) was evaluated using a univariate linear regression analysis. In order to reduce the multicollinearity caused by high degree of correlation between femur neck aBMD and femur total aBMD (Pearson’s R = 0.864), the univariate regression analysis was performed after excluding one independent variable (femur total aBMD). Each variable with a significant outcome in the univariate linear regression analysis (*P* < 0.10) was integrated into the multivariate linear regression to determine the associated factors of HGS. In the multivariate linear regression analysis, the significance level was set at *P* < 0.05 (two tails). All statistical analyses were performed using the SPSS software (ver. 23.0; SPSS Inc., Chicago, IL, USA).

## Results

### Demographic and clinical parameters and bone properties

The average age of the participants at the initial outpatient clinic visit was 75.21 ± 9.37 years (range: 62–101 years), and the average BMI was 24.06 ± 3.35 (range: 16.38–33.92). Sixty participants had an injury on their dominant side (**Table 1**). The average Ct of the radial forearm was 1.58 ± 0.20 mm (range: 1.00–2.35 mm) and the average Cd was 1445.22 ± 126.41 HU (range: 1053.13–1705.99 HU). The average HGS was 20.2 ± 5.9 (range: 6.9–35.0) (**Table 2**).

**Table 1:** Demographic characteristics of the participants.

Characteristics	Number or Score
**Participants**	108
**Mean age at diagnosed (years)**	75.21 (62–101)
**Height (cm)**	153.8 (138.4–166.5)
**Weight (kg)**	56.3 (38.6–74.3)
**Affected side (Right / Left)†**	52 (48.1%) / 56 (51.9%)
**Whether the dominant hand is affected side (Yes / No)†**	59 (54.6%) / 49 (45.4%)

* Descriptive values are shown as mean ± standard deviation (range of values) or number of cases (proportion)†.

**Table 2:** Clinical parameters and the bone properties of the participants.

Characteristics		Number or Score
**Lumbar total (L1-L4)**	aBMD (g/cm^2^)	0.82 (0.55–1.26)
	T-score	- 2.12 (- 5.0–0.4)
**Femur neck**	aBMD (g/cm^2^)	0.60 (0.31–0.86)
	T-score	- 2.41 (- 5.2 –- 0.3)
**Femur total**	aBMD (g/cm^2^)	0.70 (0.29–0.98)
	T-score	- 1.78 (- 5.4–0.5)
**Ct of radial forearm bones (mm)**		1.58 (1.00–2.35)
**Cd of radial forearm bones (HU)**		1445.22 (1053.13–1705.99)
**Hand grip strength (kg)**		20.2 (6.9–35.0)

* Descriptive values are shown as mean ± standard deviation (range of values).

aBMD, areal bone mineral density; Ct, cortical thickness; Cd, cortical density; HU, hounsfield unit.

### Comparison of bone qualities between the patients with lower HGS and those with higher HGS

Results obtained from the independent t-test showed that the significant differences of age, the femur aBMDs, and the Ct and Cd of the radial forearm bones between the two groups (**Table 3**). However, no statistical significance of BMI and lumbar aBMD was detected among the two groups.

**Table 3:** Results obtained from the independent t-test between the patients with lower HGS and those with higher HGS.

	Patients with lower HGS	Patients with higher HGS	P value
**Number of patients**	38	70	N/A
**Average HGS**	13.76 ± 2.87	23.67 ± 3.84	N/A
**Age**	82.81 ± 9.82	72.64 ± 6.89	< 0.01
**BMI**	23.96 ± 3.89	23.77 ± 3.11	0.79
**Femur neck aBMD**	0.54 ± 0.13	0.62 ± 0.09	< 0.01
**Femur total aBMD**	0.63 ± 0.13	0.74 ± 0.10	< 0.01
**Lumbar total aBMD**	0.80 ± 0.16	0.83 ± 0.11	0.24
**Ct of radial forearm bones (mm)**	1.45 ± 0.22	1.65 ± 0.23	< 0.01
**Cd of radial forearm bone (HU)**	1356.05 ± 137.48	1493.62 ± 88.82	< 0.01

* Descriptive values are shown as mean ± standard deviation.

N/A, not applicable; HGS, hand grip strength; BMI, body mass index; aBMD, areal bone mineral density; Ct, cortical thickness; Cd, cortical density; HU, hounsfield unit.

### Associations between handgrip strength and the independent variables

The univariate analytical results showed that the Ct of the distal radius (*P* < 0.001), the Cd of the distal radius (*P* < 0.001), the age (*P* < 0.001), the height (*P* < 0.001), the weight (*P* = 0.082), and the femur neck aBMD (*P* < 0.001) were significantly associated with HGS (**Table 4**). The six variables were included in a multivariate linear regression analysis, which showed that the increase in HGS was associated with a higher Cd of the distal radius (*P* < 0.001) as well as height (*P* = 0.004) (**Table 5**). In addition, the Ct of the distal radius, as well as the weight, the age, and the femur aBMD were not significantly associated with HGS.

**Table 4:** Univariate linear regression analysis of factors related to handgrip strength.

Associated factors	Regression coefficient	Standard error	95% Confidence interval	*P* value
Age	- 0.359	0.050	(-0.459, -0.259)	< .001*
Height	0.387	5.477	(0.246, 0.663)	< .001*
Weight	0.168	5.856	(-0.017, 0.272)	.082*
Affected side	1.824	1.130	(-0.416, 4.065)	.109
Whether the dominant hand is affected side	1.869	1.136	(-0.383, 4.121)	.103
Lumbar total aBMD	6.872	4.273	(-1.600, 15.344)	.111
Femur neck aBMD	19.631	4.773	(10.167, 29.094)	< .001*
Ct of distal radius (mm)	10.420	2.130	(6.198, 14.642)	< .001*
Cd of distal radius (HU)	0.030	0.003	(0.023, 0.037)	< .001*

*P < 0.1 by Univariate linear regression analysis.

aBMD, areal bone mineral density; Ct, cortical thickness; Cd, cortical density; HU, hounsfield unit.

**Table 5:** Multivariate linear regression analysis of factors related to handgrip strength.

(R^2^ = 0.507, *P* value < 0.001)					
Associated factors	Unadjusted		Standardized		*P* value
	B	SE	*β*	t	
Cd of distal radius	0.022	0.004	0.475	4.992	< .001*
Ct of distal radius	-0.439	2.635	-0.018	-0.167	.868
Age	-0.124	0.064	-0.197	-1.930	.056
Femur neck aBMD	1.329	5.046	0.025	0.263	.793
Height	0.270	0.090	0.230	2.983	.004*
Weight	0.008	0.058	0.011	0.143	.887

*P < 0.05 by Multivariate linear regression analysis.

aBMD, areal bone mineral density; Ct, cortical thickness; Cd, cortical density; HU, hounsfield unit.

## Discussion

The forces of muscles applied to the insertion sites are critical to the maintenance of bone integrity [30]. The interactions between the forearm flexor muscles and the bone properties of the forearm cortical bone might be postulated in this manner. Several studies have reported the associations between HGS and systemic BMD [31], and one study was conducted with the attempt to understand the relationship between HGS and the properties of the focal cortical bone in patients with rheumatoid arthritis [32]. However, due to a small sample number and the limitation of including only arthritic, it was difficult to determine the relationship between HGS and the properties of the focal cortical bone. Therefore, the objective of the present study was to investigate the association between HGS and the properties of the radial forearm cortical bone in a large subset of patients.

The parameters of the present study were measured based on 3D reconstructed CT models of patients with simple type DRF without severe articular comminution, metaphyseal comminution, as well as ulnar fracture. Therefore, a relatively constant ROI was used for each participant based on 3D masks adjacent to the origin of the flexor pollicis longus and the flexor digitorum superficialis, which are the muscles responsible for the production of HGS [29, 33]. Bones are known to adapt to their functional loads by altering their geometry and microstructures [27]. The sustained strain of muscles could elicit the activation of the mechanosensitive osteocytes and osteoblasts, leading to changes in bone architecture [34]. The results of the present study showed a significant association between the focal cortical bone density of the radial forearms, where the flexor muscle is originated, and HGS. Thus, it can be concluded that the properties of the cortical bones of the radial forearm could be changed through local and mechanical bone-muscle interactions.

The aforementioned results revealed that patients with lower HGS had relatively low cortical bone quality and femur aBMDs compared to those with higher HGS. Moreover, HGS is determined by the functions of extrinsic flexor muscles, which are mostly originated from the forearm cortical bones [35] and can be strengthened through hand exercises. Considering the mutual dependence of muscle activities and bone metabolism [15], it can be speculated that the strengthening of HGS could result in the improvement of the cortical bone properties of the radial forearm bone. Consequently, the strengthening of the distal forearm bone can lead to a decreasing occurrence of fractures. Further studies are needed to evaluate whether the improvement of HGS can enhance the property of the forearm bone and reduce the occurrence of DRF.

Our results showed that Ct was significantly associated with HGS in the univariate analysis but lost its significance during the multivariate analysis. Nakamura et al. reported a significant association between the Ct of the distal radius and HGS in Japanese patients with type 2 diabetes [36]. However, their study adopted only a univariate statistical method the analysis of the relationships between those two factors. The Ct could be affected by the skeletal size of the individuals [37]. Considering the mutual dependence between height, weight, and Ct, the statistical significance of Ct and weight was inevitably lost in the multivariate analysis. Therefore, even though associations were present for the Ct in relation with HGS, these associations seemed to be less than in case of the Cd as the Ct could be affected by other demographic features of the individuals, including height, weight, and ethnicity excluding HGS.

The reconstructed 3D CT images used in the present study could provide relatively reliable and sufficient information on cortical bone qualities in comparison with those of the microCT or the pQCT. Moreover, there is no association with either additional radiation exposure or medical costs as the cortical bone properties can be analyzed using a 3D reconstruction modeling software based on conventional CT data. Owing to the rapid development in graphic processing computer devices, including graphic processing units, the time need for the 3D reconstruction using personal desktops has been remarkably reduced [38]. With technological advancement, the 3D CT modeling software can be operated intuitively through a user-friendly interface in a way that physicians and surgeons, who are non-specialists in 3D image processing, can easily reconstruct target sites into 3D images and can subsequently perform accurate image analysis.

This study had several limitations. First, HGS values in this study were estimated using the 10% rule, which might differ from the actual grip power. Secondly, 850 HU was used as the threshold of the dense cortical bone based on previous studies; however, the HU value of the dense cortical bone may vary among participants. Thirdly, as the data were derived from female patients with DRF, there is no information provided by the present study about gender-specific differences. Fourthly, as the variations in the origin of flexor muscle may have existed among the participants, the ROI in this study may not reflect the actual muscle origin of each participant. Fifthly, due to the cross-sectional study design, causal relationships between HGS and the cortical bone quality could not be derived. Sixthly, in addition to cortical thickness and cortical density, other important parameters, including cortical bone cross-sectional area or cortical bone cross-sectional perimeter could not be evaluated in this study. Finally, only participants with DRF were included in this study, therefore the general population might not be accurately represented. Future studies including healthy individuals of both genders would be required.

## Conclusions

The aforementioned results showed that HGS was associated with the properties of the cortical bone of the radial forearm, which could support the theory of mechanical muscle-bone interactions. In addition, the 3D conventional CT reconstruction programs used in this study could be one of the useful modalities for the evaluation of cortical bone properties.
